# A person-based approach to emotion socialization in toddlerhood: Individual differences in maternal emotion regulation, mental-health and parental sense of competence

**DOI:** 10.1038/s41598-023-40850-x

**Published:** 2023-08-21

**Authors:** Gizem Arikan, Asiye Kumru

**Affiliations:** Department of Psychology, Ozyegin University, 34794 Istanbul, Turkey

**Keywords:** Human behaviour, Psychology

## Abstract

Mothers adopt various emotion socialization strategies and sometimes exhibit contradictory responses. Thus, it is essential to understand how mothers differentiate in their use of emotion socialization strategies, and whether a set of emotion socialization responses is associated with individual differences in emotion regulation, mental health, and parental sense of competence during toddlerhood. Therefore, we used a person-centred approach to identify mothers’ emotion socialization responses and then compared mothers based on the aforementioned characteristics. The mothers (N = 680) with toddlers (M = 23.56 months) responded to the Coping with Toddlers’ Negative Emotions Scale, the Emotion Regulation Questionnaire, the Brief Symptom Inventory, and the Parental Sense of Competence Scale. The 3-profile-solution revealed: Unspecified (moderate scores in all emotion socialization strategies), supportive (high scores in supportive emotion socialization strategies) and mixture profiles (high in all emotion socialization strategies). The supportive and mixture profiles scored highly in cognitive reappraisal. Unspecified and mixture profiles did not vary in expressive suppression and mental health symptoms, but they scored lower than supportive profile mothers. In the parental sense of competence, the supportive profile scored higher than the mixture profile. The results showed mothers mainly using supportive emotion socialization strategies can demonstrate adequate emotion regulation and benefit from psychological well-being that potentially boosts parenting competence.

## Introduction

Socialisation can be defined as a general process by which the members of society pass on the ways of their thinking, behaving, and displaying emotions to the next generation^1^. Starting from the early years, a child experiences, observes, evaluates, learns emotional expressions and responses from his/her caregiver^2,3^. In this sense, a set of cultural practices conveying the modes and strategies to express and regulate emotions within social interactions carry great importance^4^. Thus, parents provide a base for the child to process emotions via emotion discussion, emotion expression, and positive or negative reactions to the emotions of the child as covered in Eisenberg et al.’s Heuristic Model^2^. In the model, parents’ responses to children’s negative emotions can affect children’s social-emotional competencies. Studies have revealed that emotion socialisation can be essential for helping children grow into emotionally and socially competent adolescents and adults^5^ which can be facilitated by interventions.

To design effective interventions in the early years, recent studies have concentrated on a person-based approach to identify the mothers utilising different emotion socialisation strategies and what distinguishes them from each other^6–9^. Mothers use different and/or contradictory emotion socialisation responses (i.e., being supportive in managing a child’s emotions and/or showing distress or punitive reactions) frequently documented in non-Western cultures^6,9^. In other words, they engage in both supportive and unsupportive emotion socialisation reactions.

Emotion socialisation is a multifaceted and complex process. Developmental challenges of mothers in setting limits, maintaining discipline, and finding adequate methods to deal with negative emotions can be critical and show inter-individual and intercultural variations^10^. These developmental challenges can also differ based on the child’s age. For example, closer assistance in tasks and allowing autonomy might be needed for early childhood, whereas, in preschool establishing control can be demanding for parents^11,12^. Still, there is little known about how parents use different combinations of emotion socialisation strategies and how these profiles of parents differ based on parental characteristics such as emotion regulation, mental health, and parental sense of competence, especially in the early years^13^. Therefore, we adopted a person-centred approach to classify mothers of toddlers with respect to emotion socialisation strategies and aimed to detect whether they differ in emotion regulation, mental health, and parental sense of competence that can reciprocally impact emotion socialisation throughout the child’s development.

Emotion socialisation strategies are examined by focusing on parents’ reactions to their child’s negative emotions that are categorised as either supportive or unsupportive reactions^2^. Comforting the child, attempting to teach methods to regulate his/her emotions and expressing them effectively are supportive reactions. Reacting with negative and self-focused emotion, using punitive or minimising methods, showing intolerance to wishes and ignoring the child exemplify unsupportive reactions^2^.

The cultural characteristics (i.e., individualism vs collectivism)^14^ can influence emotion socialisation through shared cultural norms. How a person expresses, displays and shares his/her emotional experience is shaped by these norms indicating what is desirable and undesirable. Thus, in parenting practices, these norms are utilised in order to supervise the child about the limits of the emotional experience and boundaries of the group. For instance, expressing emotions openly is widely welcomed in Western cultures while not showing emotions or limiting emotional expression to sustain societal harmony can be desirable in the non-western collectivist cultures^15^. Expressive encouragement, dominantly used in individualistic cultures, seemed to be less adaptive compared to minimization reactions in Chinese culture^15^. Although supportive strategies are highly valued in the literature dominated by studies from individualistic and Western cultures, non-western research showed that supportive strategies could be either lacking or accompanied by unsupportive strategies. A recent study with Korean mothers revealed that only 19.7% of them mainly used supportive strategies^6^. Similarly, Travethan et al. demonstrated nearly half of the young adolescents’ mothers in India and China adopted moderately adaptive (i.e, utilising emotion-focused and culturally salient training-oriented strategies at the same time) and diffused (i.e., utilising all strategies moderately at the same time) strategies^9^.

Furthermore, modernisation, urbanisation, and embracing the Western lifestyle endow changes in emotion socialisation or may result in using a mixture of different emotion socialisation strategies that is noted both between and within cultures^16–18^. For example, in semi-structured interviews, Turkish mothers reported using different emotion socialisation strategies and engaging minimization strategies preceded by positive and negative messages^19^. In a more recent cross-cultural study recruiting highly educated mothers, Turkish mothers of toddlers engaged in mixed regulatory strategies in a delayed-gratification task depicting distraction, warmth, and negative control^17^. However, toddlerhood research in non-Western cultures carrying distinct features such as Turkish culture, represented with collectivistic and Islamic characteristics^20–22^, is still rare.

The findings above suggest that mothers can experience distinct features in emotion socialisation that can be revealed in their preferences of emotion regulation strategies as well. Gottman et al. proposed that parents’ own skills and capacities could influence parenting behaviour but there is a lack of research examining how parents’ own emotion regulation relates to their parental emotion socialisation behaviours^23^. For example, low maternal emotion regulation and cognitive regulatory capacity (i.e., avoiding distractions, paying attention, and setting priorities) are linked with punitive and ineffective parenting strategies as well as lack of monitoring, and involvement^13^. Therefore, it is worth noting that parents’ own emotion regulation is one of the parenting capacities and is prominent in multiple parenting models^24^.

The process of emotion regulation involves how an individual influences his/her emotions, when and how the person experiences, and exhibits emotions^25^. There are two distinct strategies, namely, cognitive reappraisal and expressive suppression. Cognitive reappraisal refers to a re-evaluation of the situation that gives rise to certain emotions and aims to ease its impact^26,27^. Hence, a person can reduce the effect of negative emotions or facilitate positive emotions. On the other hand, expressive suppression targets preventing the expression of emotion by suppressing it^26,27^.

The research demonstrated that cognitive reappraisal is positively associated with using praise in interactions^28^, supportive emotion socialisation^29^ and can moderate maternal anger towards young children^30^, while, emotional suppression is negatively related to parental guidance, warmth and responsiveness in parent–child interactions^31^. In a recent study, cognitive reappraisal was positively and expressive suppression was negatively associated with supportive strategies (i.e., coaching) in Japanese mothers of 2–5-year-old children^32^. In the same study, both emotion regulation strategies and mental health symptoms were related with emotion socialisation. Thus, Japanese mothers’ depression level was positively associated with less coaching and anxiety level was associated with more unsupportive socialisation (i.e., dismissing). Further, both cognitive reappraisal and suppression acted as mediators for the relationship between psychological symptoms and emotion socialisation in a sample of parents with 3–10-year-olds^33^. Although studies indicated specific relationships between emotion regulation strategies and parenting, we may still detect cultural differences in the relationship between emotion regulation and mental health outcomes^34,35^.

Contrary to expectations, the meta-analysis of Hu et al. demonstrated that cognitive reappraisal and expressive suppression can be differentially related with mental health indicators in the Eastern and Western cultures^35^. Since the use of emotion regulation strategies and their relationship with psychological well-being can show variations in Eastern cultures compared to Western cultures, maternal emotion regulation strategies may also exhibit differences in Turkish mothers. Parallel to this, in the study of Arens et al. with German and Turkish women, it was shown that Turkish women without mental health problems scored high both in cognitive reappraisal and expressive suppression, representing emotion regulation balance unlike healthy German women endorsing cognitive reappraisal highly^34^. Similarly, in a sample of mothers with children reported both cognitive reappraisal and suppression at the same time^36^. Hence, little is known about how mothers utilising different emotion socialisation strategies vary in their use of emotion regulation strategies. It would be important to enlighten if mothers using both supportive and non-supportive emotion socialisation strategies can show characteristics of emotion regulation balance as underscored before^34^. In this regard, the psychological well-being of the mothers can be also linked with parental emotion socialisation strategies during toddlerhood.

World Health Organization^37^ defines mental health as “a state of well-being in which the individual realises his or her own abilities, can cope with the normal stresses of life, can work productively and fruitfully, and is able to make a contribution to his or her community”. Not experiencing psychiatric symptoms such as anxiety, depression, somatization, obsessive–compulsive disorder and hostility can also indicate maternal mental health^38–40^. In the early years of childhood, maternal mental health may shift to a deteriorated state affecting the mother–child relationship and parenting^41^. In line with that, mothers’ capacity to deal with children’s emotions and facilitate effective emotion regulation strategies can be hard, and this can result in impaired emotion socialisation.

A study with mothers of toddlers showed that maternal depression was positively associated with unsupportive emotion socialisation and this association persisted for 1 year^42^. In the same study, maternal emotion regulation behaviours moderated the relationship between depression and granting wish strategies, an unsupportive emotion socialisation response^42^. Besides depression, other psychological symptoms can also contribute to problems in emotion socialisation. In the Breaux et al. mothers with symptoms of anxiety, personality disorders, and substance use showed more unsupportive reactions towards their children’s negative affect^43^. Similarly, maternal depression, anxiety and hostility positively predicted unsupportive emotion socialisation in Turkey during toddlerhood^44^. This underlines the importance of collecting reports of maternal symptoms in the early years of childhood. In addition to maternal mental health, mothers using emotion socialisation responses can also differ regarding their sense of confidence in their parenting skills and abilities.

Parent’s confidence in their parenting capacity can influence parent–child interactions^45^ and child-rearing practices^46^ throughout the development^47,48^. Thus, parental sense of competence involves the expectation of a parent on his/her ability to care and parent his/her child as well as his/her efficiency and contentment in the parenting role^49–52^. Besides, Sanders et al. emphasised the role of the self-regulatory mechanism as the underlying element for the parental sense of competence entailing self-sufficiency, parental efficiency, self-management, and personal agency^53^. Self-sufficiency refers to being resourceful, resilient, and independent in parenting responsibilities. Parental self-efficacy involves a high level of self-efficacy and positive expectations for change a parent can achieve. Self-monitoring, determination in goals and having criteria to evaluate himself/herself are related to self-management. Making self-attributions about a child’s positive change indicates personal agency. Hence, Sanders et al.’s framework for parental sense of competence involves both cognitive and emotional facets that can reflect on mother-child interaction as well as emotion socialisation reactions in the early years^53^.

The study of Slagt et al. revealed that mothers’ sense of competence predicted positively supportive parenting, referring to involvement and warmth, and negatively inept discipline (i.e., showing anger, and irritability) towards elementary school children ^54^. There is also evidence in samples with smaller children and their mothers^52^. In a recent study, parental sense of competence positively predicted positive parenting encompassing warmth, structure, and autonomy support, and negatively predicted negative parenting including rejection, coercion, and chaos in toddler-mother interactions during a 20-min-lab procedure^55^. Although there is a wide array of research on the contribution of parental sense of competence to the parent–child relationship, whether the parental sense of competence does differ in mothers using different emotion socialisation strategies is not fully addressed in the literature. Thus, in the Heuristic Model of Socialisation, parental characteristics carry an important role in emotion-related parenting practices^13^. Hence, adopting a person-centred approach to understand how parental characteristics of emotion regulation, mental health, and parental sense of competence can vary based on emotion socialisation strategies of mothers in toddlerhood can expand our understanding for person-tailored interventions.

Therefore, the aim of the study was to test whether maternal characteristics of emotion regulation, mental health symptoms and parental sense of competence explain mothers’ emotion socialisation characteristics. First, we explored how mothers of toddlers differ in their use of emotion socialisation reactions to toddlers’ negative emotions via a person-based approach, latent profile analysis (LPA). We expected to find at least three profiles (a) a supportive profile indicated by high problem-focused and emotion-focused responses, relatively lower expressive encouragement, distress, granting wish and punishment responses, (b) an unsupportive profile indicated by low scores on problem-focused responses, emotion-focused responses, high scores on punishment, distress, granting wish and minimization responses, (c) a mixture profile indicated by moderate to high scores across all maternal emotion socialisation responses. Second, we expected mothers in supportive profile would score lower on expressive suppression, and mental health symptoms while scoring higher on cognitive reappraisal and parental sense of competence compared to unsupportive and mixture profiles. We also expected mothers in unsupportive and mixture profiles did not differentiate from each other on expressive suppression, cognitive reappraisal, mental health symptoms and parental sense of competence.

## Results

### Analysis strategy

The analysis is divided into two parts. In the first part, we adopted a person-based approach to identify emotion socialization profiles of mothers and ran a series of Latent Profile Analysis (LPA) in MPlus 8.0^55^. The descriptive statistics for emotion socialization strategies derived from coping with toddlers’ negative emotions scale were as follows: Emotion-focused reactions (Min = 3.82, Max = 7, M = 6.11, SD = 0.67); problem-focused reactions (Min = 3.58, Max = 7, M = 6.04, SD = 0.71); distress reactions (Min = 1.31, Max = 6.31, M = 4.19, SD = 0.90); punitive reactions (Min = 1.33, Max = 7, M = 3.48, SD = 1.19); expressive encouragement reactions (Min = 1, Max = 7, M = 4.57, SD = 1.43); minimization reactions (Min = 1.08, Max = 7, M = 5.03, SD = 1.07) and granting wish reactions (Min = 1.20, Max = 6.90, M = 3.95, SD = 1.01). To compare models, we adhered to the following fit indices: The Akaike information criterion (AIC^56^), the Bayesian information criterion (BIC^57^), and the sample-size-adjusted BIC (SSA BIC^58^), better models represented with lower scores. Relative entropy^59^ ranging between 0 and 1 is also taken into consideration, higher scores indicate more accuracy. Further, the Parametric Boostrapped Likelihood Ratio Test^60^, and Adjusted Lo- Mendell-Rubin Likelihood Ratio Test (A-LMR^61^ were used to detect improvement between models (*p* < 0.05). In the second part, we compared mothers in emotion regulation strategies of cognitive reappraisal and expressive suppression, mental health, and parenting sense of competence by conducting a series of one-way ANOVAs and ANCOVAs for different profiles. Tukey HD was referred for post-hoc comparisons. When the homogeneity of variance assumption for ANOVA is violated Welch’s test and Games-Howell post-doc comparison are reported.

### Data analyses

Before the analysis, univariate and multi-varied outliers were handled according to Tabachnick and Fidel^62^) in SPSS 27^63^. There were 12 multivariate outliers eliminated from the further data analysis. The data were checked for normality based on criteria for skewness and kurtosis^64^. The skewness values varied between − 1.15 and 0.04, and kurtosis values varied between 1.64 and 0.19 showing that the variables were normally distributed. There were 8 systematic missing cases that were excluded from the profile analysis.

In the first part, we examined 1 to 4 profile solutions (see Table 1) based on the criteria above and the 3-profile solution was the best fitting. The first profile named unspecified, consists of 93 mothers, 14.1% of the sample. This group does not crystallise in their emotion socialisation strategies since they report moderate scores in all emotion socialisation responses. The second profile, a supportive group, is characterised by high scores in emotion-focused and problem-focused reactions whereas low scores in distress, punitive, and granting wish (n = 213, 32.3%). The third and final profile is called mixture. It has high scores both in supportive strategies of emotion-focused and problem-focused reactions as well as distress, punitive, expressive encouragement, and minimization reactions (n = 354, 53.6%). Please see Table 2 for the means and standard deviations of three profiles for emotion socialisation reactions.

**Table 1:** Latent Profile Analysis (LPA) models’ fit indices and group assignment accuracy.

Model	BIC	AIC	Entropy	saBIC	LMR-LRT	Model comparison	BLRT
1 Profile	12,833.21	12,770.30	–	12,788.76	–	–	–
2 Profile	12,305.61	12,206.75	0.92	12,235.77	< 0.001	1 vs 2	< 0.001
3 Profile	12,011.36	11,876.55	0.77	11,916.11	< 0.001	2 vs 3	< 0.001
4 Profile	11,882.02	11,711.26	0.80	11,761.37	ns	3 vs 4	ns

*BIC* Bayesian information criterion, *AIC* akaike information criterion, *saBIC* sample size adjusted Bayesian information criterion, *LMR-LRT* Lo-Mendell-Rubin likelihood ratio test, *BLRT* bootstrap parametric likelihood ratio test, *ns* nonsignificant.

**Table 2:** Means and standard deviations of emotion socialization reactions with respect to profiles.

Mean (SD)							
Profiles	Emotion focused reactions	Problem focused reactions	Distress reactions	Punitive reactions	Expressive encouragement reactions	Minimization reactions	Granting wish reactions
1 Profile (Unspecified)	4.95 (0.61)	4.72 (0.56)	4.07 (0.69)	3.76 (1.01)	4.02 (0.97)	4.39 (0.83)	4.02 (0.75)
2 Profile (Supportive)	6.24 (0.49)	6.34 (0.45)	3.53 (0.89)	2.48 (0.73)	4.34 (1.57)	4.40 (1.17)	3.13 (0.80)
3 Profile (Mixture)	6.32 (0.43)	6.21 (0.43)	4.61 (0.66)	4.00 (1.07)	4.86 (1.37)	5.58 (0.67)	4.43 (0.85)

**Figure 1:**
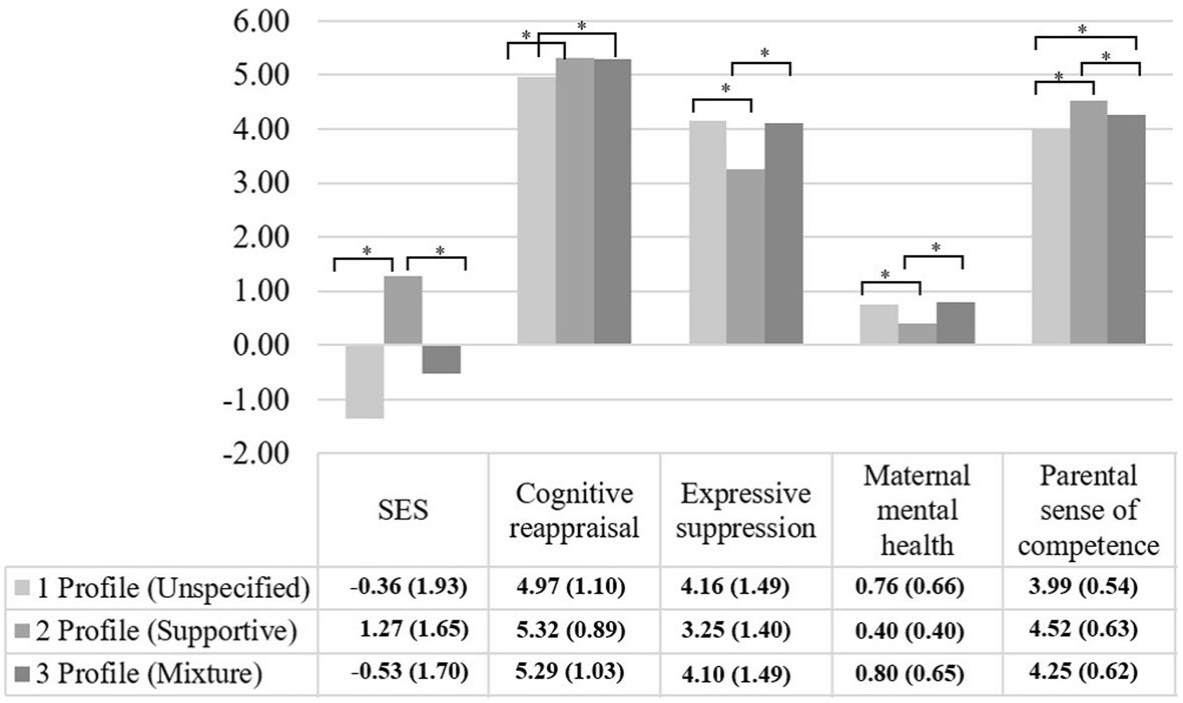
The means and standard deviations of profile solutions.

The second part involves profile comparisons for emotion regulation strategy of cognitive reappraisal, expressive suppression, mental health and parenting sense of competence. Before conducting comparisons, we examined the bivariate correlations of demographic factors of child’s age and maternal SES with variables. The child’s age was not correlated to any of the outcome variables except for parental sense of competence, r = 0.09, *p* < 0.05. Maternal SES was negatively correlated with emotional suppression r = − 0.32, *p* < 0.001; and maternal mental health r = 0.24, *p* < 0.001. We assigned these demographic characteristics as covariates in the relevant ANCOVAs. Homogeneity of variance assumption was violated for emotion regulation strategy of cognitive reappraisal, and mental health symptoms. The groups did vary for cognitive reappraisal, Welch’s F(2, 237,97) = 67.51, *p* < 0.001 and expressive suppression after controlling for SES, F(2,653)=9.28, *p* < 0.001 (See Fig. 1).

For cognitive reappraisal, profile 1 (unspecified profile) and 2 (supportive profile) differed. Profile 1 and 3 (mixture profile) also differed significantly. But there was no difference between profile 2 and profile 3. For expressive suppression strategy, profile 1 and profile 2 differed from each other. Similarly, profile 2 was different from profile 3. But no difference was detected between profile 1 and profile 3. The mothers varied in maternal mental health as well, Welch’s F(2, 240.45) = 46.50, *p* < 0.001. Based on Games Howell post-hoc test, profile 1 and profile 2 were different. Profile 2 and 3 differed from each other but not profile 1 and profile 3. After controlling for the child’s age there was a group difference in parental sense of competence, F(2,655) = 25.28, *p* < 0.001. Profile 1 and Profile 2 were different. Profile 1 and Profile 3 were different, Profile 2 and Profile 3 were also different.

## Discussion

The first aim of the present study was to explore the emotion socialisation profiles of Turkish mothers. Our findings partially supported the first hypothesis that the majority adopted supportive strategies as in the supportive profile or could score high in both supportive and non-supportive strategies as in the mixture profile. Rather than the unsupportive profile, we found a third profile, namely unspecified, in which mothers score moderately in all emotion socialisation strategies and do not crystallise in their strategy preferences. Reporting both supportive and non-supportive strategies can be detected mainly in non-Western cultures^6,9,65^ rather than in Western cultures^66,67^.

In the study of Choi and Kang^6^, mothers with relatively older children (7–12-year-olds) represented with five profiles, restrained (average scores problem-focused, emotion-focused and expressive encouragement, and lower scores in minimization, punitive and distress responses), ineffective (low scores in supportive responses and average scores in non-supportive responses), harsh (high scores in non-supportive and low scores in supportive responses), dismissive (average scores for supportive responses, and high scores in minimization, punitive and distress), and supportive (high scores in expressive encouragement, emotion-focused and problem-focused). However, in that study, one of the statistical indicators, LMR-a LRT, becomes non-significant for the 4-profile solution. This makes the 5-profile solution questionable and requires considering the 4-profile solution. Another study with Chinese fathers yielded a 4-profile solution, supportive (low in punitive, moderate in minimization and high in supportive reactions), balanced (moderate in combined emotion/problem-focused score and non-supportive, below average in expressive encouragement), disengaged (low in all emotion socialisation reactions) and harsh (low in supportive and high in unsupportive reactions)^65^. When Indian and Chinese mothers of young adolescents are considered, the three-factor- solution with adaptive (high supportive responses except for expressive encouragement, and low in non-supportive responses except for minimization and punitive responses), moderate (high in emotion-focused, problem-focused, training-oriented, experience-oriented, and low in scolding, no talking) and diffused (average scores in all) profiles were revealed^9^. The variation across samples may derive from age differences of the children because emotion socialisation strategies of the parents may change and adapt to the developmental needs (i.e., rigid boundaries during adolescence). Moreover, the emotion socialisation measures were either modified (i.e., inclusion of new dimensions such as training-oriented, and scolding) or did not yield the same factor structure with the Coping with Toddlers’ Negative Emotions Scale^68,69^ in these studies. However, in our research we used the same factor structure of the Coping with Toddlers’ Negative Emotions Scale and mothers of toddlers reported three compositions of emotion socialisation strategies, unspecified; mixture; and supportive profiles.

The second hypothesis was confirmed by our findings indicating there were differences in cognitive reappraisal, expressive suppression, mental health symptoms, and parental sense of competence among emotion socialisation profiles. Namely, the mothers of toddlers in an unspecified profile used less cognitive reappraisals than supportive and mixture profiles. This shows mothers’ tendency to differentiate not only in emotion socialisation but also in cognitive reappraisal, an adaptive coping that is associated with both positive parental and child outcomes^28,30^. Contrary to expectations, mothers in supportive and mixture profiles scored similarly in cognitive reappraisal. This highlights mothers scoring relatively high in supportive emotion socialisation are likely to engage in adaptive coping strategies and cognitive reappraisal. Parallel to our findings, Cabecinha- Alati et al.^29^ showed that maternal cognitive reappraisal is positively associated with supportive responses and negatively related to unsupportive responses of mothers with 8-to-12-year-olds. Further, Chinese fathers adopting high-to-moderate supportive emotion socialisation strategies did also report higher cognitive reappraisal than those predominantly utilising unsupportive strategies^65^. However, there was no significant difference in expressive suppression among these profiles^65^. Contrarily, in our study supportive mothers reported less expressive suppression unlike mixture and unspecified profiles. This may indicate lack of suppression can be positively associated with supportive emotion socialisation in the toddlerhood years and mothers’ skills in emotion regulation can further their parenting abilities and enhance their capacity to manage toddlers’ negative emotions effectively. Still, it is critical to underline that mothers in supportive and mixture profiles did not differentiate in cognitive reappraisal strategies. Thus, using high supportive strategies might be uniquely associated with cognitive reappraisal and using unsupportive strategies may not be essential for these mothers.

In addition to the research pinpointing the association between emotion regulation strategies and mental health^35^, our study revealed that mothers with different emotion socialisation profiles could vary in their mental health symptoms. The mothers of toddlers in the supportive profile scored less in mental health symptoms than in unspecified and mixture profiles. Previously, Lee et al.^70^ demonstrated difficulties in maternal emotion regulation, which is positively linked with a lack of supportive emotion socialisation in mother-preschooler dyads. Moreover, the studies conducted with parents of adolescents^71^, and toddlers^44^ revealed the importance of parental symptomatology. Depressive symptoms of parents with adolescents predicted emotion socialisation responses in the 5-month follow-up^71^. In a sample of mothers with toddlers, maternal depression, anxiety, and hostility positively predicted the unsupportive emotion socialisation of mothers^44^. Hence, lack of symptoms and effective emotion regulation strategies seems to contribute to maternal self-regulatory capacity^47^ which is likely to be manifested in parental sense of competence as well.

In our study, the mothers in the supportive profile reported higher parental sense of competence unlike mothers in the mixture and unspecified profiles. Among all profiles, the unspecified profile scored the lowest in the parental sense of competence demonstrating a set of emotion socialisation strategies can provide mothers with the relevant toolbox to manage their toddlers’ emotional reactions. Further, using all strategies in moderation may not be helpful for parenting. The moderate scores in emotion socialisation strategies might be an indicator of indecisiveness in parenting that can be associated with caregiving helplessness, and permissive or enmeshed parenting^72,73^. Thus, Toz et al.^74^, showed that maternal caregiving helplessness is associated mainly with anxiety and expressive suppression. Similarly, in our sample, mothers in the unspecified profile scored highly in both expressive suppression and symptoms. Therefore, we may infer that such mothers might experience caregiving helplessness and lack of parental competence mutually serving challenges in emotion socialisation. Moreover, maternal depression can moderate the permissive parenting style^75^, and maternal mental health problems can be linked with permissive parenting and hostility^76^. These findings can help us to understand how the mothers in the mixture and unspecified profiles may have lack of parental competence. Both their tendency to use less effective emotion regulation strategies and experiencing more mental health problems may jeopardise their parenting skills and result in caregiving helplessness and permissive parenting.

The findings revealed three distinct emotion socialisation profiles and mothers mainly using supportive emotion socialisation strategies can demonstrate adequate emotion regulation and benefit from psychological well-being that may potentially boost their parenting competence. Although the majority of the mothers in the study scored highly in supportive emotion socialisation strategies, nearly half of the mothers use both supportive and unsupportive emotion socialisation strategies to the same extent. These mothers can engage in cognitive reappraisal as supportive mothers do. But they fail to score low in expressive suppression, and symptoms, and high in parental sense of competence, unlike supportive mothers. Therefore, they might be prone to experience further parental and child-related problems in the future. This may require the attention of professionals conducting interventions.

Our study is one of the first that focuses on the toddlerhood period by applying a person-based approach in a non-Western and predominantly Islamic country. However, there are several limitations of the study. First, we recruited only mother reports. Other caregivers (i.e., fathers) can be influential in the emotion socialisation process and should be considered in the future. Second, the cross-sectional design does not allow us to follow whether mothers’ emotion socialisation profiles change over time. Jordan^77^ showed that parents with children in different age groups do vary in their depression and stress. Thus, following the toddlerhood period, maternal characteristics may be altered by both internal and external dynamics. This can potentially result in the use of different emotion socialisation strategies in varied degrees. Also, the Coping with Children’s Negative Emotions Scale can be criticised due to acquiring an individualistic and western framework in evaluating parental responses disregarding cultural differences. Hence, other assessment techniques such as interviews^78^ may provide a better understanding about culture’s role in emotion socialisation. Finally, there could be other factors contributing to emotion socialisation such as social support and child’s temperament that might be relevant for examining the variations in emotion socialisation profiles. Child’s characteristics such as temperament^79^ and bi-directional nature of the mother-child interaction can shape the emotion socialisation process^11,80^ throughout the development. For example, in the bidirectional or transactional models of parenting and temperament, parents’ efforts aiming to reduce a child’s negative affect and behaviours might elicit more negative affect and behaviours, which in turn might lead to negative parenting^81^. Thus, children with mothers adopting consistent strategies are less likely to experience internalization problems^66^. In line with that, research from both human and animal subjects demonstrated that predictability is a fundamentally important factor that can not only have a detrimental biological impact but also lead to psychological issues throughout the course of development^83–85^. Furthermore, a child’s negative affect and behaviours might result in negative parenting, which in turn endangers emotional regulation issues and behavioural dysregulation as well. Thus, future studies should include child outcomes of cognitive and socio-emotional functioning that can be captured with relevant screening tools and scales (i.e., Ages and stages Questionnaire^85^ and Bayley Scales of Infant and Toddler Development^86^). Due to the interactive and dynamic nature of the mother-toddler relationship future research needs can recruit dyadic observations in which identifying the use of emotion socialisation strategies in action may expand our understanding about the profiles we revealed. By addressing these issues, both researchers and clinicians can design effective intervention programs and facilitate maternal consistency in emotion socialisation strategies^82–84^. Further, studies may also consider following up mothers starting from early years and examine a wider array of aforementioned factors to identify what leads to alterations in emotion socialisation profile and maternal emotional well-being as well as parental and child characteristics.

## Methods

After receiving approval from the ethics committee of the university (Human Research Ethics Board of Ozyegin University), the study was advertised in pharmacies, community family-health centres, preschools, research lab’s social media accounts, and with the help of our graduate and undergraduate students. The inclusion criteria for the study were having no medical condition, for both the mother and toddler and being the biological mother. In-home visits conducted by graduate and/or undergraduate psychology students, mothers gave written consent (illiterate mothers’ consents were gathered from legally authorized representatives) and filled out 45-minute-long questionnaire packs in a counterbalanced format. Graduate or undergraduate students read the questions to illiterate mothers. At the end, mothers were thanked with a toy or a pack of diapers and a play activities booklet for toddlers. All methods are carried out in accordance with relevant guidelines.

Six hundred eighty mothers (M = 31.69 years, SD = 4.811, Age Range: 18–47) with toddlers (51.8% male, M = 23.56 months, SD = 6.99, Age Range: 12–38) participated in the study. The majority of the mothers were married (97%). Only 0.7% of the mothers were illiterate and 1.6% of the mothers can only read and write. The distribution of education level was as follows: 14.9% of primary school graduates, 15.4% of secondary school graduates, 24.6% of high school graduates, 34.1% of college or university graduates and 8.7% had a master’s degree or a PhD. The distribution of household income was as follows: 1.5% had an income of 850TL and below, 17.2% had an income between 851 and 1500 TL, 31.9% had an income between 1501 and 3000 TL, 14.1% had an income of 5001–7500 TL and 18.1% had an income of 7501 and above. When the study was initiated the poverty threshold for a 4-member family was 4997 TL^87^. The lowest maternal education level and household income were coded as 1 and incremented by 1 point. Z-scores of education level and household income were taken and their mean score consisted of socioeconomic status (SES) for mothers.

### Measures

#### Demographic form

The form consisted of questions involving maternal education history, total household income, maternal age, marital status, and child’s age.

#### Emotion socialization

Coping with Toddlers’ Negative Emotions Scale^68,69^ consists of 12 hypothetical scenarios and assesses 7 emotion socialisation strategies of a parent with a 7-point Likert response scale (1 = very unlikely; 7 = very likely). The scale yielded 7-factor structure with adequate internal consistency in a sample of low SES Turkish mothers of toddlers^88^: Emotion-focused reactions (α = 0.80); problem-focused reactions (α = 0.81); distress reactions (α = 0.74); punitive reactions (α = 0.84); minimization reactions (α = 0.75); expressive encouragement (α = 0.91) and granting wish reactions (α = 0.67). In the present study the Cronbach alpha values were as follows: Emotion-focused reactions (α = 0.83); problem-focused reactions (α = 0.83); distress reactions (α = 0.78); punitive reactions (α = 0.84); minimization reactions (α = 0.85); expressive encouragement (α = 0.92) and granting wish reactions (α = 0.72).

#### Emotion regulation

The emotion regulation questionnaire was developed by Gross and John^89^ and consists of ten items with 7-point Likert scale (1 = strongly disagree; 7 = strongly agree). It has two subscales, cognitive reappraisal and expressive suppressions and has adequate internal consistencies with 0.88 and 0.82 alpha values, respectively^90^. In the present study, Cronbach alpha values were 0.78 for cognitive reappraisal and 0.75 for expressive suppression.

#### Mental health

The Brief Symptom Inventory developed by Derogatis^91^ and a 53-item version shorter version with a 5-point Likert-type scale (0 = not at all; 4 = extremely) was used^57^. The scale includes nine subscales measuring psychological and physiological complaints in the past week. The internal consistency values varied from 0.71 to 0.85^92^ and in the Turkish version 0.76 and 0.90, respectively^93^. In the present study, a total score was used with a Cronbach alpha of 0.97.

#### Parental sense of competence

The parental sense of competence scale (PSOC)^94,95^ measures parental self-esteem with sixteen items. The scale uses a 6-point Likert-type response (1 = strongly disagree; 6 = strongly agree). Cronbach alpha of the scale was 0.79. The Turkish version has a high internal consistency as well with a 0.88 Cronbach alpha value^96^. In the present study, a total scale was used with a Cronbach Alpha of 0.74.
